# AEBP1 promotes epithelial-mesenchymal transition of gastric cancer cells by activating the NF-κB pathway and predicts poor outcome of the patients

**DOI:** 10.1038/s41598-018-29878-6

**Published:** 2018-08-10

**Authors:** Jun-yan Liu, Lei Jiang, Jia-jia Liu, Tao He, You-hong Cui, Feng Qian, Pei-wu Yu

**Affiliations:** 1 Department of General Surgery and Center of Minimal Invasive Gastrointestinal Surgery, Southwest Hospital, Third Military Medical University, Chongqing, 400038 China; 2 Department of Oncology Surgery, The First Hospital of Lanzhou University, Lanzhou, 730030 China; 3 Institute of Pathology and Southwest Cancer Center, Southwest Hospital, Third Military Medical University, Key Laboratory of Tumor Immunopathology of Ministry of Education of China, Chongqing, 400038 China

## Abstract

Adipocyte enhancer binding protein 1 (AEBP1) is a transcriptional repressor that plays a critical role in regulating adipogenesis. Recent studies have indicated that AEBP1 might function as a candidate oncogene and is overexpressed in several human malignancies. However, the role of AEBP1 in gastric cancer (GC) remains largely unknown. This study aimed to investigate the expression pattern, prognostic significance and biological function of AEBP1 in human gastric cancer and to explore the underlying mechanism. We found that both the mRNA and protein levels of AEBP1 were significantly increased in human GC tissues. Elevated AEBP1 expression was significantly correlated with poor overall survival in patients with both early-stage (Tumor, Node, Metastases (TNM) TNM I and II) and late-stage (TNM III and IV) GC. Silencing AEBP1 markedly suppressed the proliferation, migration, invasion, metastasis and epithelial-mesenchymal transition of GC cells. Moreover, we demonstrated that knockdown of AEBP1 in GC cells led to inhibition of the NF-κB pathway by hampering the degradation of IκBα. Thus, AEBP1 might be served as a promising prognostic indicator and a potential therapeutic target in human GC.

## Introduction

Although the incidence and mortality of gastric cancer (GC) have steadily declined within recent decades in nearly all populations^1^, GC remains the fourth most commonly diagnosed cancer and the third most common cause of cancer-associated mortality worldwide, particularly in East Asian countries^2,3^. Currently, despite the progress made in early diagnosis and multimodal treatment strategies that enhanced the survival of patients with GC, the prognosis of patients with advanced-stage GC remains poor, with a five-year survival rate of 20%^1,2^. Furthermore, the survival outcomes of GC patients with distal metastases are even worse, with a median survival time of one year^4^. Invasion and metastasis are the key biological characteristics of GC cells, which are responsible for the high mortality rate in patients with GC^5^. However, the molecular mechanisms underlying GC invasion and metastasis have not been fully elucidated. Therefore, it is imperative to identify novel therapeutic targets and explore the underlying mechanisms regarding GC invasion and metastasis, which would contribute to identifying novel therapeutic approaches and developing effective targeted treatments for patients with GC.

Adipocyte enhancer binding protein 1 (AEBP1) was originally identified as a transcriptional repressor that negatively regulates adipogenesis^6^. Recent studies have demonstrated that AEBP1 showed higher transcription activity in patients with nonalcoholic steatohepatitis and played an important role in the pathogenesis of nonalcoholic fatty liver disease^7^. Upregulation of AEBP1 was discovered in Alzheimer’s disease and promoted the progression of the disease^8^. AEBP1 was found to be a novel candidate gene for the pathogenesis of Ehlers-Danlos syndrome^9^. Moreover, AEBP1 played a critical role in regulating the proinflammation process in macrophages, including macrophage cholesterol homeostasis, foam cell formation and the development of atherosclerosis^10,11^.

Notably, recent studies have illustrated an important role of AEBP1 in tumorigenesis and tumor progression. AEBP1 is upregulated in glioma cells^12^ and in breast^13^, bladder^14^, and serous ovarian cancers^15^, as well as in vemurafenib-resistant melanoma cells^16^. However, the expression, prognostic significance and function of AEBP1 in GC remain unknown.

In the present study, we showed that the mRNA and protein expression of AEBP1 was upregulated in GC tissues and cell lines. High expression of AEBP1 was associated with poor overall survival in patients with both early-stage (Tumor, Node, Metastases (TNM) I and II) and late-stage (TNM III and IV) GC. Furthermore, we demonstrated that knockdown of AEBP1 impaired the proliferation, migration, invasion, metastasis and epithelial-mesenchymal transition (EMT) of GC cells by attenuating the degradation of IκBα, leading to inhibition of the NF-κB pathway. Our results suggest that AEBP1 might be considered as a promising prognostic indicator and potential therapeutic target in patients with GC.

## Results

### AEBP1 is highly expressed in human GC tissues and cell lines

**Figure 1:**
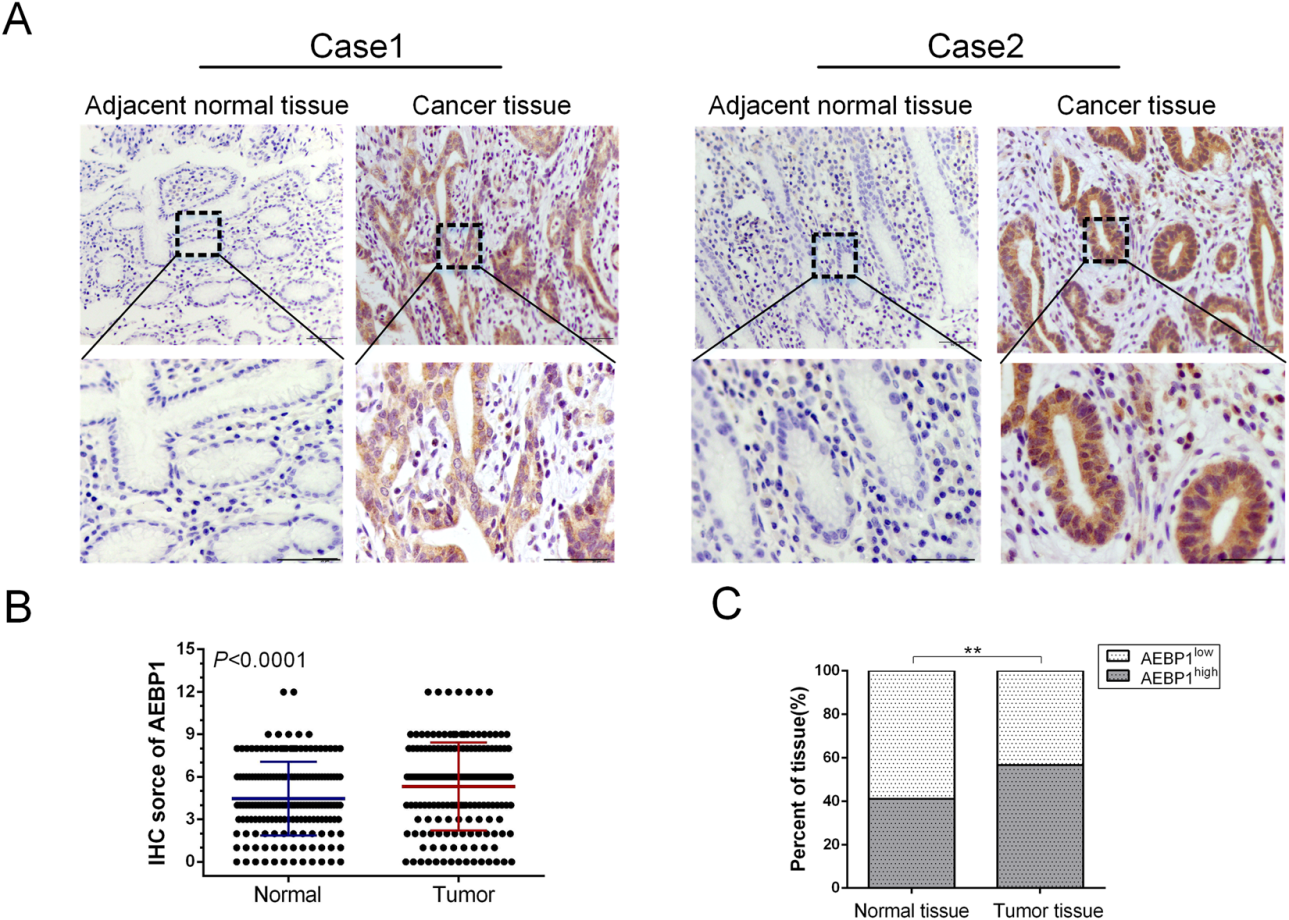
AEBP1 is highly expressed in gastric cancer tissues. (**A**) Representative immunohistochemical staining of AEBP1 in adjacent normal mucosa and gastric cancer tissue. Bar, 50 μm. (**B**) The immunohistochemical score of AEBP1 was significantly higher in gastric cancer tissues than in adjacent normal tissues. (**C**) High expression of AEBP1 was more frequent in gastric cancer tissues than in adjacent normal tissues; **P < 0.01.

**Figure 2:**
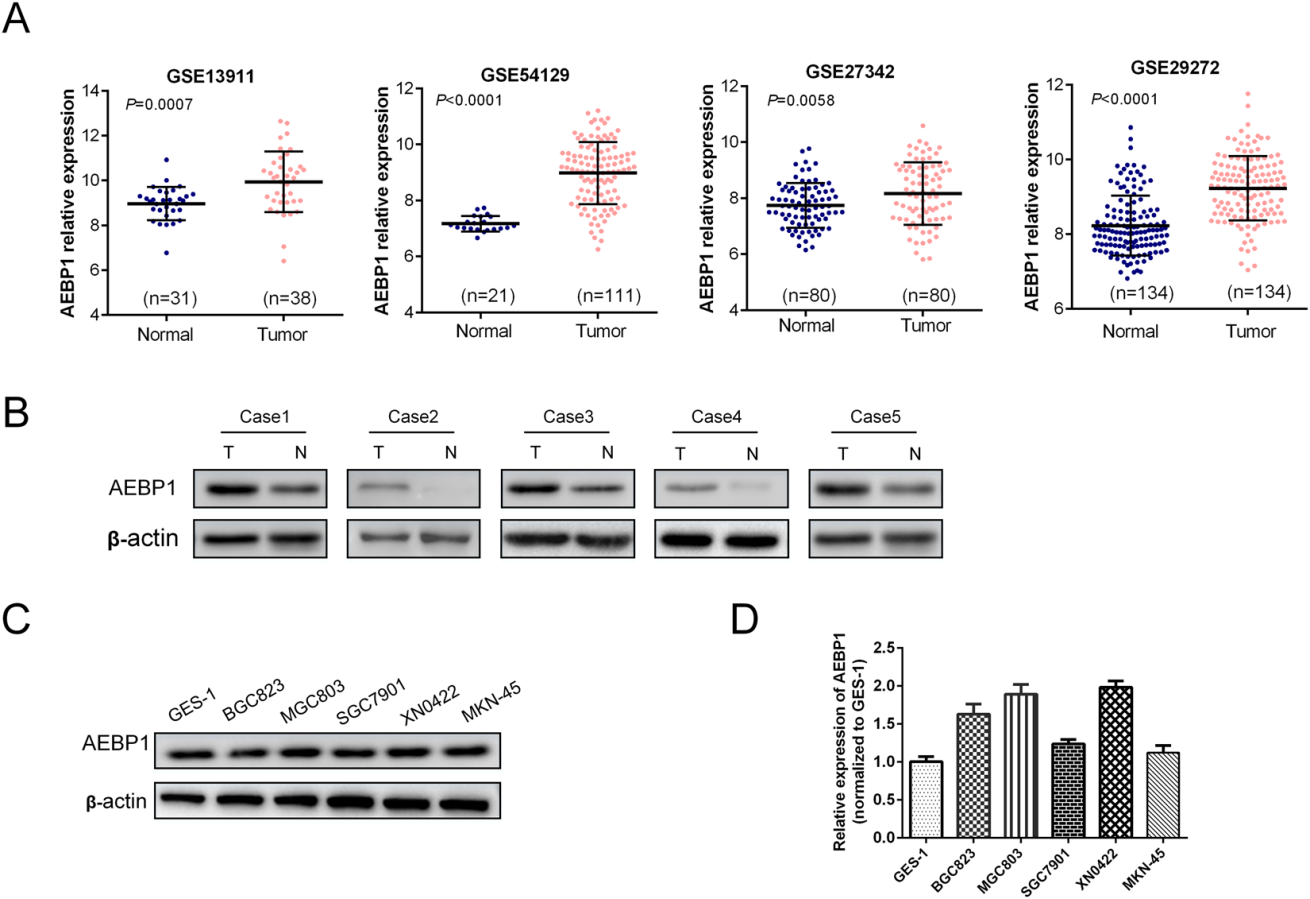
Expression of AEBP1 is elevated in gastric cancer tissues and cell lines. (**A**) Analyses of the AEBP1 mRNA levels from the NCBI GEO datasets GSE13911, GSE54129, GSE27342 and GSE29272. (**B**) Western blotting analysis of AEBP1 expression in 5 fresh surgical gastric cancer specimens (T) and corresponding adjacent normal tissues (N). (**C** and **D**) Western blotting and qRT-PCR analyses of AEBP1 in four gastric cancer cell lines (MKN-45, BGC823, MGC803, SGC7901) and a primary gastric cancer cell XN0422 as well as in GES-1. The western blots were derived under the same experimental conditions from the same cell lysates; the original full-length Western blot images are shown in Supplementary Fig. S3.

The expression of AEBP1 was examined in 166 paired samples of GC tissues and corresponding adjacent normal tissues by immunohistochemistry (IHC) and H&E staining (Supplementary Fig. S1A). We observed that the IHC score of AEBP1 was markedly increased in GC tissues compared with that in adjacent normal tissues (P < 0.001, Fig. 1A and B). Moreover, the high expression percent of AEBP1 was significantly higher in GC tissues (56.63%, 94/166) as compared with that in adjacent normal tissues (40.96%, 68/166) (P = 0.004, Fig. 1C). We further detected the mRNA expression of AEBP1 by analyzing the NCBI GEO database, the results of GSE13911 (P = 0.0007), GSE54129 (P < 0.0001), GSE27342 (P = 0.0058) and GSE29272 (P < 0.0001) datasets all demonstrated that the mRNA levels of AEBP1 were significantly higher in GC tissues than in normal tissues (Fig. 2A). We also examined the expression of AEBP1 in five pairs of fresh GC tissues and corresponding adjacent normal tissues by Western blotting analysis, and the results showed that AEBP1 was upregulated in GC tissues compared with that in adjacent normal tissues (Fig. 2B). Furthermore, we detected the expression of AEBP1 in four GC cell lines (BGC823, MGC803, SGC7901, MKN-45) and a primary gastric cancer cell XN0422 (established in our laboratory) as well as in the immortalized gastric epithelium cell line GES-1. Our results indicated that both the mRNA and protein levels of AEBP1 were upregulated in GC cells compared with those in GES-1 (Fig. 2C and D). Because the expression of AEBP1 was relatively higher in MGC803 and XN0422 cells, they were used in subsequent experiments. Taken together, these results indicate that AEBP1 is upregulated in GC tissues and cell lines, suggesting that AEBP1 might function as an oncogene in GC.

### Elevated expression of AEBP1 is correlated with clinicopathological features and associated with poor prognosis of patients with GC

**Figure 3:**
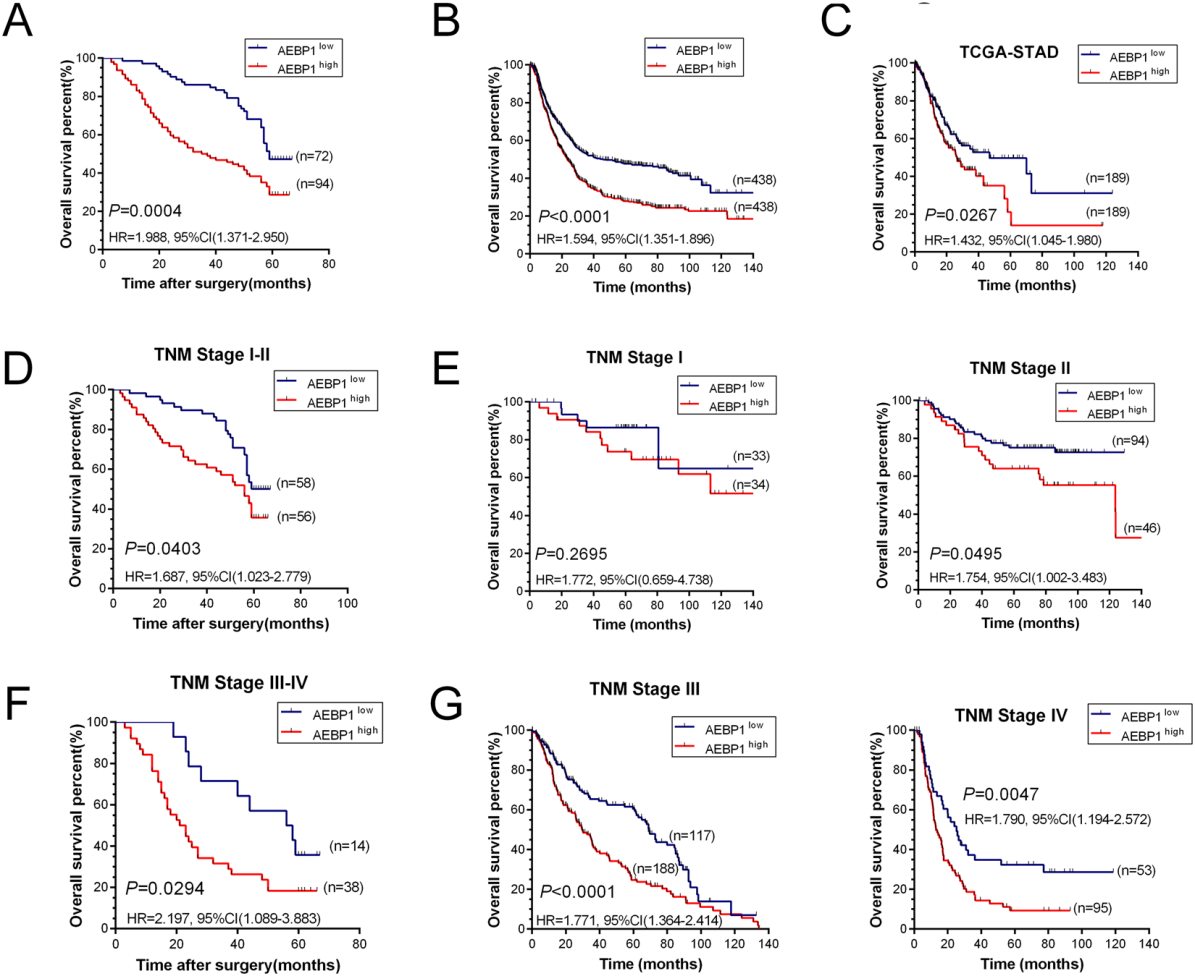
Elevated expression of AEBP1 is associated with poor overall survival (OS) rate in patients with both early-stage (TNM I and II) and late-stage (TNM III and IV) gastric cancer. (**A**) Kaplan-Meier analysis of 166 patients with gastric cancer indicating high AEBP1 expression is associated with poor overall survival (P = 0.0004). (**B**) Kaplan-Meier analysis of 876 patients with gastric cancer from NCBI GEO database revealing AEBP1^high^ patients had a significantly lower overall survival rate than those with AEBP1^low^ tumors (P < 0.0001). (**C**) Kaplan-Meier analysis of 378 patients with gastric cancer from TCGA-STAD database indicating AEBP1^high^ patients had a lower overall survival rate than those with AEBP1^low^ tumors (P = 0.0267). (**D**) Kaplan-Meier analyses of patients with TNM I and II stage gastric cancer from our cohort showing high expression of AEBP1 is correlated with a lower overall survival rate (P = 0.0403). (**E**) Kaplan-Meier analyses from the NCBI GEO database in patients with TNM I (P = 0.2695) and TNM II (P = 0.0495) stage gastric cancer. (**F**) Kaplan-Meier estimation of patients with TNM III and IV stage gastric cancer from our cohort showing high expression of AEBP1 is correlated with a lower overall survival rate (P = 0.0294). (**G**) Kaplan-Meier analyses from NCBI GEO database in patients with TNM III (P < 0.0001) and TNM IV (P = 0.0047) stage gastric cancer.

We investigated the relationship between the clinicopathological features and AEBP1 expression in patients with GC. As shown in Table 1, upregulation of AEBP1 was positively correlated with T stage (P = 0.005), N stage (P = 0.005) and TNM stage (P = 0.004), but not with sex (P = 0.504), age (P = 0.453), tumor location (P = 0.603) or histological grade (P = 0.950). Moreover, we explored the prognostic significance of AEBP1 in patients with GC. During a median follow-up period of 56 months (range, 3–67 months) in our cohort of 166 patients with GC, 105 patients (63.25%) died, and 61 (36.75%) survived. Kaplan-Meier survival analysis revealed that patients with AEBP1^high^ GC (n = 94) had a shorter OS than those with AEBP1^low^ tumors (n = 72, HR = 1.988, 95% CI (1.371–2.950), P = 0.0004, Fig. 3A). Moreover, we validated the prognostic significance of AEBP1 in two larger cohorts, using the NCBI GEO and TCGA databases. The results from the NCBI GEO database was explored using the online tool KM plotter, and the results demonstrated that high mRNA levels of AEBP1 predicted significantly lower OS rates in 876 patients with GC (HR = 1.594, 95% CI (1.351–1.896), P < 0.0001, Fig. 3B). A similar trend was also found in the TCGA-STAD dataset, which included 378 patients with GC (HR = 1.432, 95% CI (1.045–1.980), P = 0.0267, Fig. 3C). We next analyzed the prognostic value of AEBP1 after further stratification by TNM stage, and the subgroup analysis of our data indicated that high AEBP1 expression was correlated with worse OS in patients with both early-stage (TNM I and II) (HR = 1.687, 95% CI (1.023–2.779), P = 0.0403, Fig. 3D) and late-stage (TNM III and IV) (HR = 2.197, 95% CI (1.089–3.883), P = 0.0294, Fig. 3F) GC. In addition, subgroup analyses of the NCBI GEO database indicated that patients with high AEBP1 expression were correlated with a significantly lower OS rate in patients with TNM stage II (HR = 1.754, 95% CI (1.002–3.483), P = 0.0495, Fig. 3E), TNM stage III (HR = 1.771, 95% CI (1.364–2.414), P < 0.0001, Fig. 3G) and TNM stage IV (HR = 1.790, 95% CI (1.194–2.572), P = 0.0047, Fig. 3G) GC. However, no significant difference was observed in patients with TNM stage I (HR = 1.772, 95% CI (0.659–4.738), P = 0.2695, Fig. 3E) GC. This might be due to the limited number of patients with TNM stage I GC. These results demonstrate that elevated expression of AEBP1 is correlated with a significantly lower OS rate in patients with both early-stage (TNM I and II) and late-stage (TNM III and IV) GC.

**Table 1:** The relationship between AEBP1 expression and clinicopathological features in patients with gastric cancer.

Prognostic variables	Number	AEBP1 expression		χ^2^	P
		Low	High		
Sex				0.446	0.504
Male	113	51	62		
Female	53	21	32		
Age (years)				0.563	0.453
≥60	63	25	38		
<60	103	47	56		
Tumor location				0.271	0.603
Proximal	59	24	35		
Distal	107	48	59		
Histological grade				0.004	0.950
G1 + G2	48	21	27		
G3	118	51	67		
T stage				7.986	**0**.**005**
T1-T2	65	37	28		
T3-T4	101	35	66		
N stage				8.068	**0**.**005**
N0	76	42	34		
N1-N3	90	30	60		
M stage				1.733	0.188
M0	157	70	87		
M1	9	2	7		
TNM stage				8.343	**0**.**004**
I + II	114	58	56		
III + IV	52	14	38

Cox proportional hazards regression was employed to evaluate the association between AEBP1 expression and all prognostic factors. In univariate analysis, tumor location (P = 0.041), T stage (P < 0.001), N stage (P = 0.001), M stage (P < 0.001), TNM stage (P < 0.001) and AEBP1 expression (P = 0.001, Table 2) were significantly associated with prognosis. Only significant univariate variables were incorporated into multivariate analysis, and the results revealed that T stage (P = 0.002), M stage (P = 0.001) and AEBP1 expression (P = 0.004, Table 2) were independent prognostic factors for OS in patients with GC. Taken together, elevated AEBP1 expression is significantly correlated with a lower OS rate in patients with GC, and it is an independent prognostic indicator.

**Table 2:** Univariate and multivariate analyses of overall survival in patients with gastric cancer.

Prognostic Variables	Univariate analysis			Multivariate analysis		
	HR	95% CI	P value	HR	95% CI	P value
Sex	0.876	0.580–1.323	0.530	—	—	—
Age	1.412	0.955–2.088	0.084	—	—	—
Histological grade	0.699	0.466–1.049	0.084	—	—	—
Tumor location	0.666	0.450–0.984	0.041	0.867	0.575–1.307	0.496
T stage	2.640	1.723–4.045	0.000	2.224	1.356–3.646	0.002
N stage	2.014	1.356–2.993	0.001	1.502	0.924–2.441	0.101
M stage	5.681	2.737–11.791	0.000	4.010	1.829–8.796	0.001
TNM stage	2.116	1.422–3.149	0.000	0.796	0.458–1.386	0.421
AEBP1 expression	1.999	1.341–2.981	0.001	1.829	1.216–2.751	0.004

### Knockdown of AEBP1 suppresses the proliferation of GC cells

**Figure 4:**
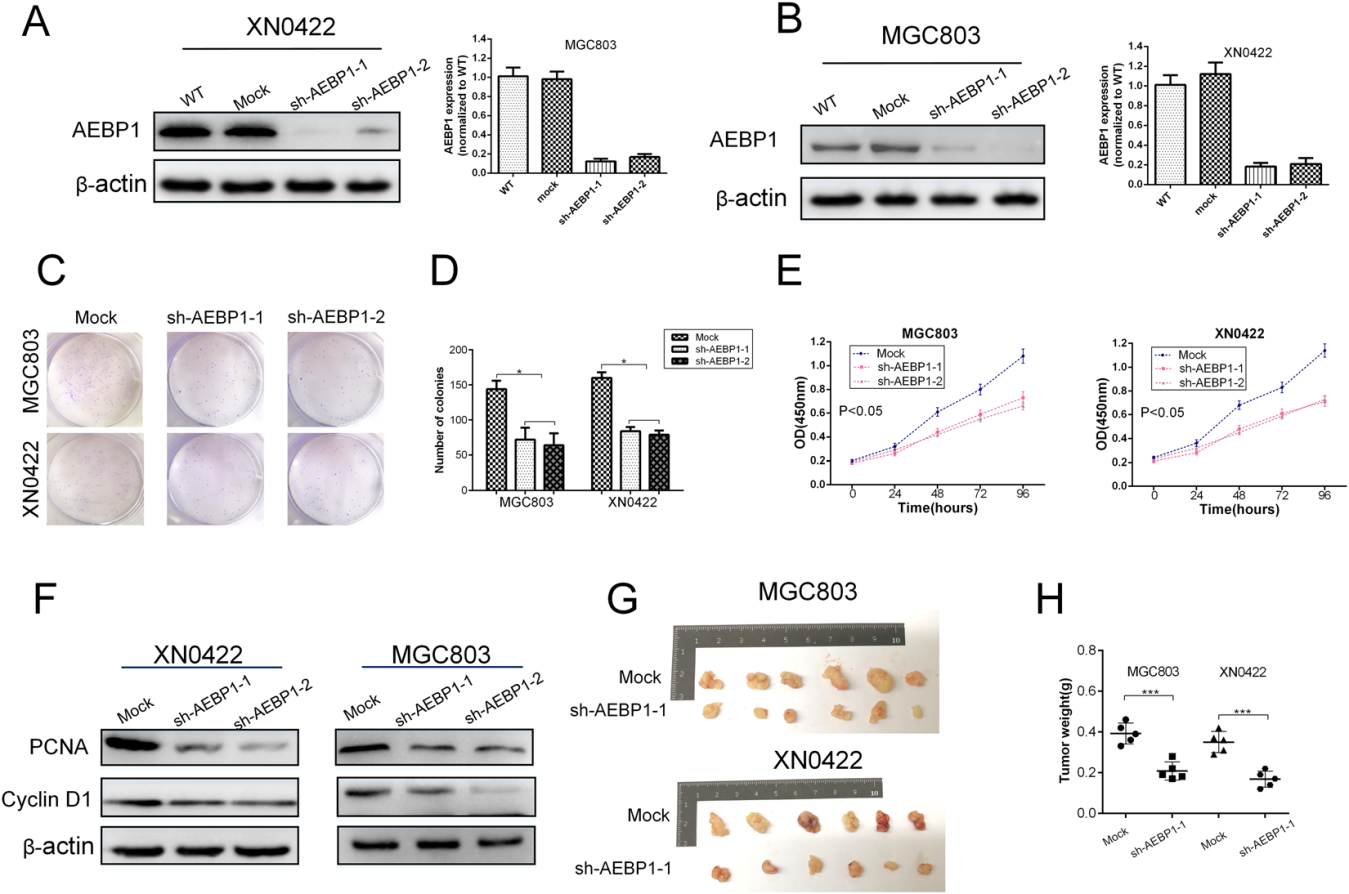
Silencing AEBP1 reduces the proliferation of gastric cancer cells. (**A**) The knockdown efficiencies of AEBP1 in MGC803 and XN0422 were confirmed by Western blotting and qRT-PCR (**B**) analyses; *P < 0.05, **P < 0.01. (**C**) Representative images of colonies formed in sh-AEBP1 and mock MGC803 and XN0422 cells. (**D**) Histograms showing the number of colonies. The means ± standard deviations from three independent experiments were shown; *P < 0.05. (**E**) CCK-8 assays showing impaired proliferation ability of MGC803 and XN0422 cells with knockdown of AEBP1. (**F**) Western blotting analyses of PCNA and cyclin D1 in AEBP1-knockdown and mock cells. (**G**) Formation of subcutaneous xenograft tumors by mock and sh-AEBP1 cells. (**H**) Analysis of the weight of xenograft tumors; ***P < 0.001. The western blots were derived under the same experimental conditions from the same cell lysates; the original full-length Western blot images are shown in Supplementary Fig. S4.

To assess the proliferative capability of AEBP1 in GC cells, CCK-8, colony formation and subcutaneous xenograft tumorigenicity assays were performed in AEBP1-knockdown and mock MGC803 and XN0422 cells. The knockdown efficiency was verified through qRT-PCR and Western blotting analyses (Fig. 4A and B). The results of the colony formation assay revealed that the number of colonies was obviously decreased in AEBP1-knockdown cells compared with that in mock cells (P < 0.05, Fig. 4C and D). In addition, the CCK-8 assays indicated that knockdown of AEBP1 markedly attenuated cell proliferation in MGC803 and XN0422 cells (P < 0.05, Fig. 4E). Moreover, the expression of two cell proliferation-related markers, cyclin D1 and PCNA, were significantly downregulated in AEBP1-knockdown GC cells (Fig. 4F). We then assessed the effect of AEBP1 knockdown on the tumorigenic potential and proliferation *in vivo* using a subcutaneous xenograft model in nude mice. The results showed that the size and weight of the xenograft tumors derived from the AEBP1-knockdown cells were significantly reduced than those of mock cells (Fig. 4G and H). These results demonstrate that knockdown of AEBP1 impairs the proliferation of GC cells both *in vitro* and *in vivo*.

### Suppression of AEBP1 impairs the migratory, invasive and metastatic capabilities of GC cells

**Figure 5:**
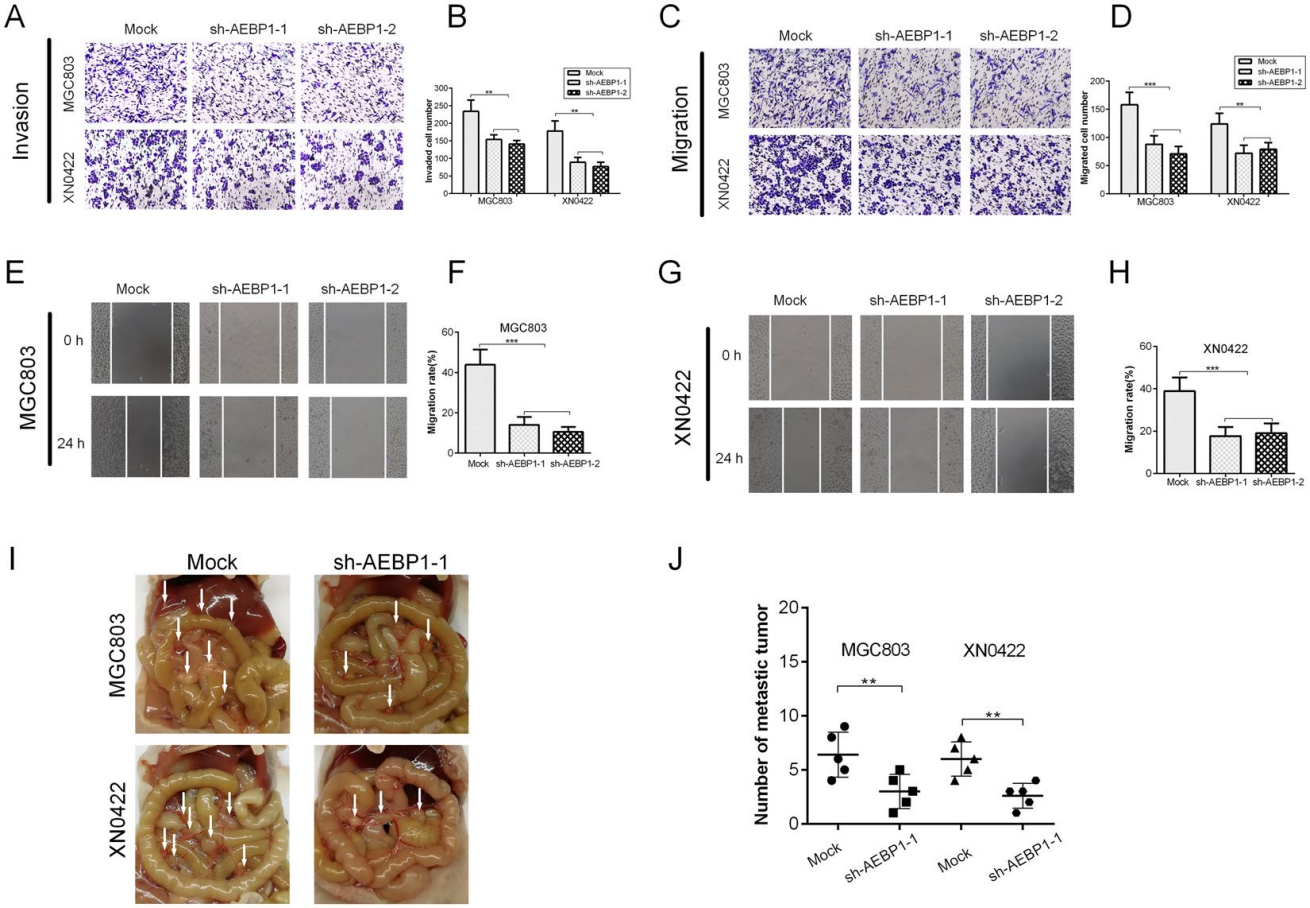
Silencing AEBP1 impairs the migratory, invasive and metastatic capabilities of gastric cancer cells. (**A**) Representative images of transwell invasion assays showing decreased invasive capabilities of GC cells with knockdown of AEBP1; magnification: 200×. (**B**) Quantification of the transwell invasion assay results; **P < 0.01. (**C**) Representative images of transwell migration assays showing decreased migratory capabilities of GC cells with knockdown of AEBP1; magnification: 200×. (**D**) Quantification of the transwell migration assay results; **P < 0.01, ***P < 0.001. (**E**) Representative images of the wound-healing assay of MGC803 indicating decreased migratory capabilities of GC cells with knockdown of AEBP1; magnification: 200×. (**F**) Quantification of the wound healing assay results from MGC803; ***P < 0.001. (**G**) Representative images of the wound-healing assay of XN0422 showing decreased migratory capabilities of GC cells with knockdown of AEBP1; magnification: 200×. (**H**) Quantification of the wound healing assay results from MGC803; ***P < 0.001. (**I**) Representative images of intraperitoneal tumor nodules formed by mock and sh-AEBP1-1 transfected MGC803 and XN0422 cells. Arrows indicate intraperitoneal nodules. (**J**) Analysis of the number of intraperitoneal nodules; **P < 0.01.

To investigate the role of AEBP1 on the migration and invasion of GC cells *in vitro*, transwell migration and invasion assays and scratch wound-healing assays were performed. The transwell invasion assay indicated that knockdown of AEBP1 significantly reduced the invaded cell number in MGC803 and XN0422 cells (P < 0.001, Fig. 5A and B). In addition, the transwell migration and wound healing assays demonstrated that knockdown of AEBP1 dramatically suppressed the migratory abilities of GC cells compared with those of mock cells (Fig. 5C–H). To assess the effect of knockdown of AEBP1 on the *in vivo* metastasis, intraperitoneal metastasis assay was performed. The results indicated that the occurrence of metastatic foci was significantly lower in nude mice implanted with sh-AEBP1-1 cells as compared with mock cells (Fig. 5I and J). These results suggest that AEBP1 contributes to the migratory, invasive and metastatic abilities of GC cells.

### AEBP1 promotes EMT of GC cells by activating the NF-κB pathway

**Figure 6:**
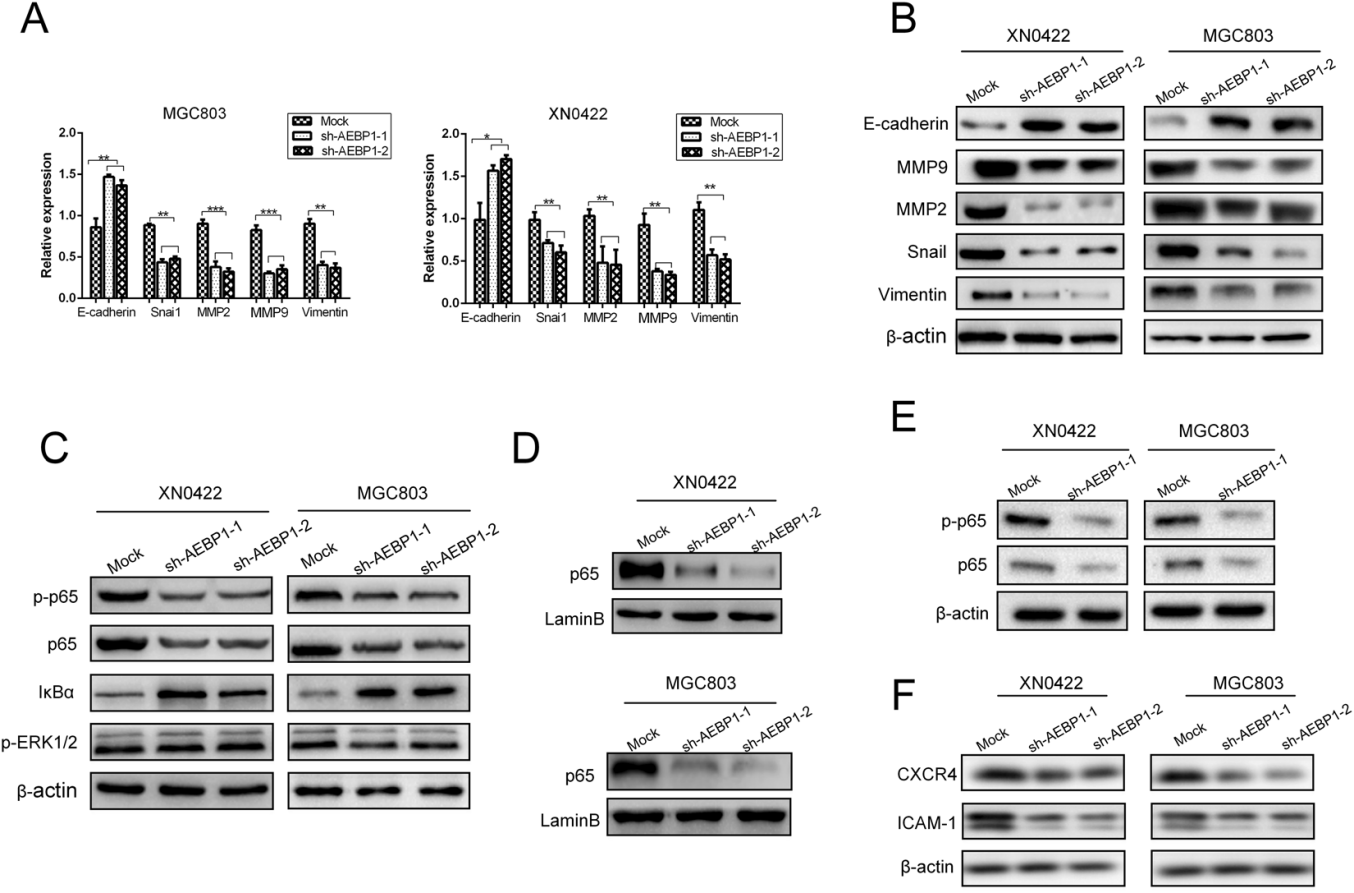
Silencing AEBP1 results in the inhibition of epithelial-mesenchymal transition (EMT) by downregulating the NF-κB pathway. (**A**) qRT-PCR and (**B**) Western blotting analyses of EMT-related markers (E-cadherin, MMP2, MMP9, snail and vimentin) in AEBP1-knockdown and mock cells; *P < 0.05; **P < 0.01, ***P < 0.001. (**C**) Protein levels of p65, p-p65, IκBα and p-ERK1/2 in AEBP1-knockdown and mock cells. (**D**) Nuclear protein expression of NF-κB p65 in AEBP1-knockdown and mock cells. (**E**) Western blotting analyses of p65 and p-p65 expression in xenograft tumors derived from the AEBP1-knockdown and mock MGC803 and XN0422 cells. (**F**) Protein levels of two NF-κB downstream targets (CXCR4 and ICAM1) in AEBP1-knockdown and mock cells. The western blots were derived under the same experimental conditions from the same cell lysates; the original full-length Western blot images are shown in Supplementary Fig. S5.

To explore the underlying mechanism of AEBP1-mediated promotion of migration, invasion and metastasis of GC cells, we assessed whether AEBP1 was involved in the regulation of EMT, which is reported to be a key process for cancer development and metastasis^17^. We selected five EMT-related markers, E-cadherin, snail, vimentin, MMP2 and MMP9, and detected the protein and mRNA levels of these markers in AEBP1-knockdown and mock MGC803 and XN0422 cells. The results indicated that the mRNA and protein expression of E-cadherin was significantly upregulated in AEBP1-knockdown cells; however, the expression of snail, vimentin, MMP2 and MMP9 were markedly downregulated (Fig. 6A and B). These observations reveal that AEBP1 promotes EMT in GC cells.

Considering that AEBP1 was reported to be involved in the regulation of NF-κB and ERK1/2 signaling pathways^16–19^, we explored the expression of NF-κB p65, phosphorylated p65 (p-p65), IκBα and phosphorylated ERK1/2 (p-ERK1/2) in AEBP1-knockdown and mock MGC803 and XN0422 cells. The results demonstrated that both p65 and p-p65 were downregulated in AEBP1-knockdown GC cells. Moreover, we found that the expression of IκBα was markedly upregulated following knockdown of AEBP1 in GC cells (Fig. 6C). However, we did not observe an apparent difference in the phosphorylation level of ERK1/2 between AEBP1-knockdown cells and mock cells (Fig. 6C). To further confirm the effects of AEBP1 on the NF-κB signaling pathway, we detected the nuclear protein expression of NF-κB p65 by Western blotting analysis, and our results demonstrated that suppression of AEBP1 significantly impaired the nuclear accumulation of p65 in both GC cells (Fig. 6D). In the xenograft tumors derived from mock and sh-AEBP1-1 GC cells, the protein expression of p65 and p-p65 were downregulated in xenograft tumors formed by AEBP1-knockdown GC cells (Fig. 6E), indicating that inhibition of AEBP1 expression resulted in impaired NF-κB activation both *in vitro* and *in vivo*. Moreover, the protein expression of two classically recognized NF-κB downstream targets, including CXCR4 and ICAM-1^20,21^ were significantly downregulated in AEBP1-knockdown GC cells (Fig. 6F).

**Figure 7:**
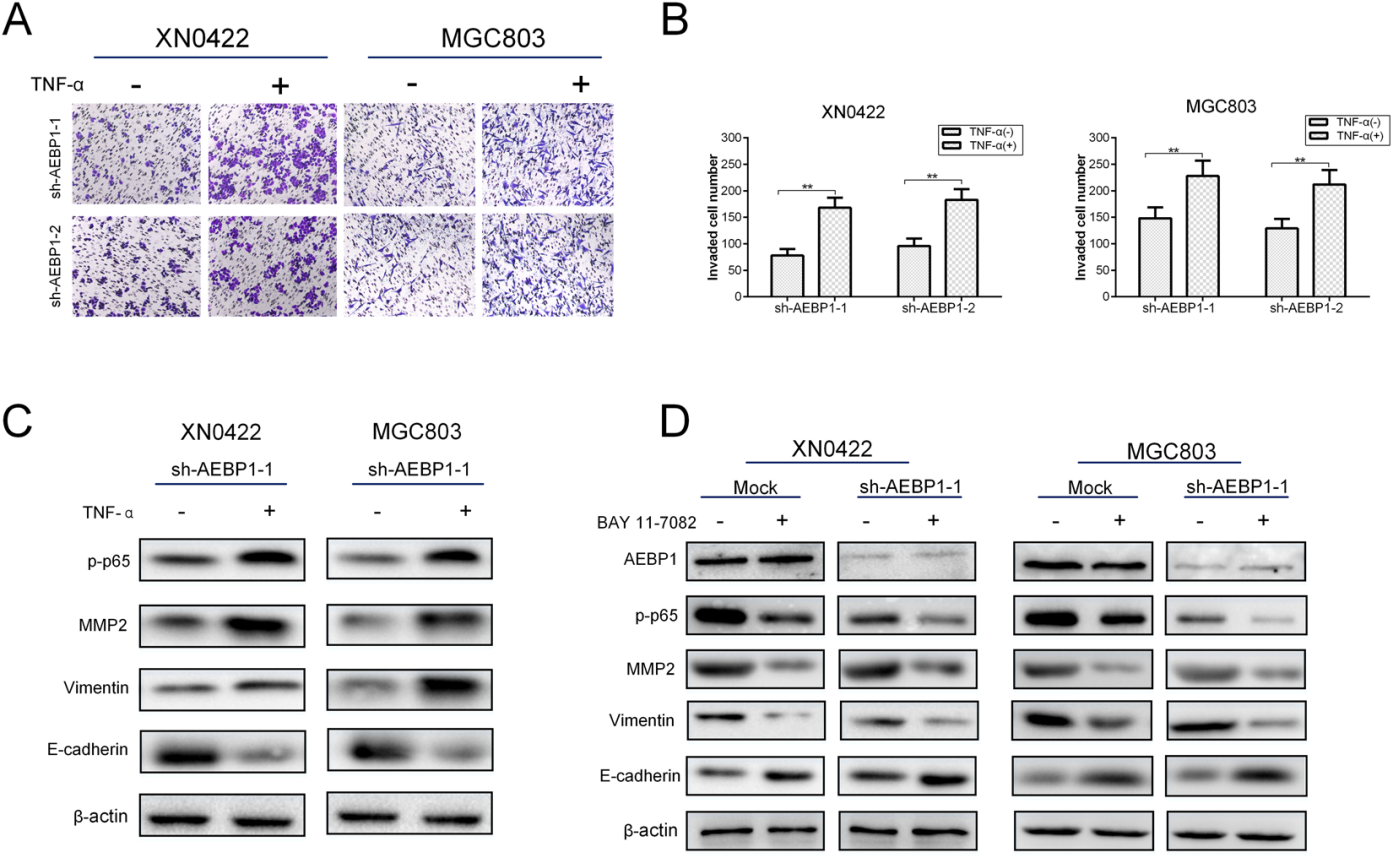
AEBP1 modulates epithelial-mesenchymal transition of gastric cancer cells by functioning as an important upstream regulator of the NF-κB pathway. (**A**) Representative images of transwell invasion assays showing TNF-α (10 ng/mL) treatment significantly reversed the impaired invasive capabilities due to knockdown of AEBP1; magnification: 200×. (**B**) Quantification of the transwell invasion assay results; **P < 0.01. (**C**) Western blotting analyses of p-p65, MMP2, vimentin and E-cadherin expression after TNF-α (10 ng/mL) treatment in mock and sh-AEBP1-1 XN0422 and MGC803 cells. (**D**) Western blotting analyses of AEBP1, p-p65, MMP2, vimentin and E-cadherin expression after BAY 11–708 treatment in mock and sh-AEBP1-1 XN0422 and MGC803 cells. The western blots were derived under the same experimental conditions from the same cell lysates; the original full-length Western blot images are shown in Supplementary Fig. S6.

TNF-α, a well-documented inducer of NF-κB signaling pathway, was used to activate NF-κB. As shown in Fig. 7A and B, activation of NF-κB by TNF-α treatment in AEBP1-knockdown GC cells significantly reversed the impaired invasive capabilities. Moreover, TNF-α treatment in AEBP1-knockdown GC cells significantly upregulated the expression of MMP2, vimentin and the phosphorylation level of p65, and downregulated the expression of E-cadherin (Fig. 7C). The results demonstrate that activation of NF-κB by TNF-α treatment effectively reverses the inhibition of EMT due to knockdown of AEBP1. To explore whether AEBP1 was an upstream regulator of the NF-κB pathway, we used a specific NF-κB inhibitor, BAY 11–7082, to suppress NF-κB activity in mock and AEBP1-knockdown GC cells. We found that BAY 11–708 significantly suppressed the expression of MMP2, vimentin and phosphorylation level of p65, and upregulated the expression of E-cadherin but did not change the expression of AEBP1 in both mock and AEBP1-knockdown GC cells (Fig. 7D), indicating that AEBP1 could promote NF-κB but could not be regulated by NF-κB. Thus, AEBP1 was an important upstream regulator of NF-κB. In conclusion, these results indicate that AEBP1 promotes EMT of GC cells through accelerating the degradation of IκBα to activate the NF-κB signaling pathway.

## Discussion

The nuclear factor kappa B (NF-κB) family is composed of five transcription factors, RelA (p65), RelB, c-Rel, NF-κB 1 (p105/p50) and NF-κB 2 (p100/p52)^22^, whose activities are regulated by inhibitor of kBa (IκBα) kinases (IKKs)^23^. The proteins form active transcription factors through homo and/or heterodimerization, and their activities are majorly regulated by the canonical and noncanonical pathways^24^. The canonical pathway consists primarily of nuclear p65/p50 and is triggered by phosphorylation of IκBα, an inhibitor of NF-κBIκB, leading to polyubiquitination and proteasomal degradation of the inhibitory protein^25^. After IκB degradation, the NF-κB subunit p65 is released and translocates from the cytoplasm to the nucleus, which activates the transcription of the target genes^26^. NF-κB transcription factor plays a critical role in the modulation of genes that regulate multiple responses in malignant cells, including cell survival, proliferation, chemoresistance, angiogenesis, inflammation and cytoskeleton remodeling^27^. The aberrant hyperactivation or upregulation of NF-κB transcription is commonly detected in cancer cells^28^. Numerous studies have highlighted the important role of NF-κB signaling in the promotion and progression of several types of malignancies, including leukemia^29^, oral squamous cell carcinoma^30^, glioblastoma^31^, and ovarian^32^, breast^33^, pancreatic^34^, prostate^35^ and gastric cancers^36^. In addition, the NF-κB pathway has been demonstrated to enhance the metastatic ability of tumor cells by transcriptionally regulating the expression of matrix metalloproteinases (MMPs), especially MMP2 and MMP9^37^, which are often detected in solid tumor tissues and are associated with tumor metastasis in many cancers, including GC. However, the modulation of the NF-κB pathway in GC remains ambiguous, and the illustration of the regulatory mechanisms of this pathway is critical to the therapeutic potential of targeting the NF-κB pathway in GC.

Previous studies have indicated AEBP1 in the regulation of the NF-κB pathway. AEBP1 was found to promote NF-κB activity in macrophages by physically interacting with IκBα through its discoidin-like domain to facilitate its phosphorylation on Ser^32^/Ser^36^ independent of IκB kinases (IKKα and IKKβ), resulting in enhanced degradation of IκBα^19^. However, the cells employed in the study were rat C6 glioma cells, peritoneal macrophages isolated from mice, and J774 macrophages (derived from mouse)^19^; it remains unclear whether AEBP1 plays a role in modulating NF-κB activity in human GC cells. In BRAF inhibitor-resistant melanoma cells, upregulation of AEBP1 mediated by hyperactivation of the PI3K/Akt-CREB (cAMP response element-binding protein) pathway activated NF-κB activity by accelerating IκBα degradation, thereby conferring acquired resistance to BRAF (V600E) inhibition^16^. In the present study, we demonstrated that both total p65 and p-p65 protein levels were downregulated in AEBP1-knockdown GC cells. Moreover, p65 protein levels in the nuclei of AEBP1-knockdown GC cells were significantly downregulated, indicating that AEBP1 played a critical role in activating the NF-κB pathway in human GC cells. Notably, the expression of IκBα was obviously upregulated in AEBP1-knockdown cells, indicative of impaired degradation resulting from downregulation of AEBP1. Our results demonstrated that AEBP1 promoted the NF-κB activity by enhancing the degradation of IκBα.

It has been demonstrated that AEBP1 could directly interact with both the phosphorylated and dephosphorylated forms of MAPK through its discoidin-like domain at the N-terminus^18^. The interaction between AEBP1 and MAPK protected MAPK from a MAPK-specific phosphatase, resulting in increased MAPK activity^18^. Hence, we detected the phosphorylation level of ERK1/2 in mock and AEBP1-knockdown GC cells. The results demonstrated that the ERK1/2 phosphorylation was not changed evidently, indicating that AEBP1-mediated invasion and migration in GC cells were mainly regulated by NF-κB signaling, not the ERK1/2 pathway.

Previous findings have indicated that AEBP1 overexpression was correlated with poor OS in patients with serous ovarian and bladder cancers^14,15,38^. AEBP1 was upregulated more than 4-fold in most primary glioblastoma multiforme (GBM) cases compared with secondary GBM^39^. In addition, silencing of AEBP1 in GBM cells led to loss of proliferative potential and apoptosis^12^. However, the expression and function of AEBP1 in GC remain unknown. Herein, we found that both the mRNA and protein levels of AEBP1 were significantly upregulated in GC tissues. Overexpression of AEBP1 was significantly correlated with poor OS in patients with both early-stage (TNM I and II) and late-stage (TNM III and IV) GC, suggesting that AEBP1 might be a promising prognostic marker in patients with GC. To our knowledge, this is the first clinicopathological study to report the relationship between AEBP1 and the clinical outcome of patients with GC.

A proteome profiling analysis has defined AEBP1 as a new EMT marker^40^. EMT is considered the first and key step for invasion and metastasis at the invasive front of GC, characterized by the loss of cellular junctions, polarity and epithelial markers, and acquisition of mesenchymal phenotype and motility^40^. As a result, epithelial markers such as E-cadherin are downregulated, and mesenchymal markers such as N-cadherin, vimentin, snail, MMP2 and MMP9 are upregulated^41^. MMP2 and MMP9 are the major enzymes that degrade the extracellular matrix (ECM) and correlate with the invasive and metastatic phenotypes of GC cells^42^. It has been reported that NF-κB is the upstream regulator of MMPs and regulates Snail expression via transcriptional and post-translational mechanisms^43–45^ and, activation of NF-κB promotes GC cell migration and invasion^46^. Our results revealed that knockdown of AEBP1 attenuated the mRNA and protein expression of mesenchymal markers, including vimentin, snail MMP2 and MMP9 with upregulation of the epithelial marker E-cadherin. These findings suggest that AEBP1 promotes the invasion, migration and metastasis of GC through enhancing EMT by activating the NF-κB signaling pathway.

In conclusion, our study illustrates an important role of AEBP1 in the malignant behavior of GC. We demonstrate that AEBP1 is highly expressed in GC tissues and cell lines, and elevated expression of AEBP1 in GC tissues is correlated with poor OS in patients with both early-stage (TNM I and II) and late-stage (TNM III and IV) GC. Furthermore, we found that AEBP1 promotes proliferation, migration, invasion, metastasis and EMT of GC cells by activating NF-κB as an upstream regulator. Thus, our results indicate that AEBP1 might be regarded as a promising prognostic biomarker and a potential therapeutic target for human GC.

## Methods

### Cells and culture

The human GC cell lines SGC7901, BGC823, MGC803, and MKN-45 and the immortalized gastric epithelium cell line (GES-1) were purchased from the Shanghai Institute of Biochemistry and Cell Biology, Chinese Academy of Sciences (Shanghai, China). The primary GC cell XN0422 was isolated and cultured in our laboratory as previously described^47,48^. Cells were cultured in RPMI-1640 medium (HyClone, USA) supplemented with 10% fetal bovine serum (FBS) (Gibco, USA) and 1% penicillin-streptomycin (Solarbio, China) at 37 °C in 5% CO_2_ and 100% humidity. Bay 11–7082 (Abcam, Cambridge, UK), a specific inhibitor of NF-κB, was used to treat cells at a final concentration of 10 μM for 12 h. For TNF-α-induced activation of NF-κB pathway, GC cells were treated with TNF-α (10 ng/mL, Sigma-Aldrich, USA) for 24 h.

### Patients and tissue specimens

GC and adjacent normal tissue samples were obtained from 166 patients with histologically confirmed gastric adenocarcinoma between January 2007 and December 2008 from the Southwest Hospital, Third Military Medical University (Chongqing, China). All patients underwent radical gastrectomy and D2 lymph node dissections and were followed up for at least 5 years. None of the patients received radiotherapy or chemotherapy prior to surgery. The clinicopathological characteristics of the 166 patients with GC are listed in Supplementary Table S1. Written informed consent was obtained from all patients enrolled into the study, and the study was approved by the Ethics Committee of Southwest Hospital. TNM stage was classified according to the 7th edition of American Joint Committee on Cancer (AJCC) staging system^49^. All data were obtained from clinical and pathologic records and included age, sex, tumor location, histological grade, depth of invasion (T stage), lymph node metastasis (N stage), distant metastasis (M stage) and TNM stage. Overall survival (OS) was defined as the time from the date of surgery to death from any cause. Five fresh tumor samples were obtained from primary GC patients who received curative surgery at Southwest Hospital from January 2017 to December 2017 and were subjected to Western blotting analysis.

### Immunohistochemistry (IHC)

One hundred and sixty-six pairs of formalin-fixed, paraffin-embedded GC tissues and corresponding adjacent normal tissues were collected and used for immunohistochemistry (IHC). Briefly, 4-μm-thick sections were cut and treated with xylene for deparaffinization and rehydration with graded ethanol. Antigen retrieval was performed by boiling in Tris-EDTA buffer (pH 9.0) in a pressure cooker for 5 min. The endogenous peroxidase activity was blocked with 3% H_2_O_2_. The sections were then blocked with normal goat serum for 30 min at room temperature and were incubated with a mouse anti-AEBP1 monoclonal antibody (1:500; Novus Biologicals, USA) at 4 °C overnight. After washing the primary antibody with PBS, a horseradish peroxidase (HRP)-conjugated secondary antibody (Dako, Glostrup, Denmark) was added, and the slides were incubated at 37 °C for 30 min according to the instructions of the Dako REAL Envision Detection System. The slides were then evaluated by two pathologists independently in a blinded manner. For the evaluation of the AEBP1 staining score, the staining intensity was scored as follows: 0, no staining; 1, weak staining; 2, moderate staining; and 3, strong staining. The percentage of positive tumor cells was scored as follows: 1 = 1–25%; 2 = 26–50%; 3 = 51–75%; and 4 > 75%. The IHC staining score was calculated by multiplying the staining intensity by the percentage of positive cells^50^. For the determination of the optimal cutoff value, IHC scores were analyzed using the statistical software X-tile^51^ (Supplementary Fig. S1B), and Youden’s index was calculated by SPSS 19.0 software (Supplementary Fig. S2). An IHC score of 4 was determined as the optimal cutoff value. Consequently, scores ≥6 were defined as AEBP1^high^, and scores ≤4 were defined as AEBP1^low^.

### Bioinformatics analysis

The mRNA expression data of AEBP1 were downloaded from NCBI Gene Expression Omnibus (GEO) (http://www.ncbi.nlm.nih.gov/geo/). Four datasets regarding GC (Accession Numbers: GSE13911, GSE54129, GSE27342 and GSE29272) were included in our study to compare the mRNA levels of AEBP1 in GC tissues and normal tissues. The mRNA expression data of AEBP1 were extracted and processed by R software 3.2.5 version (www.r-project.org, R Foundation for Statistical Computing, Vienna, Austria) and the Bioconductor project package (http://www.bioconductor.org/)^52^. The expression data of AEBP1 were transformed with log2 and normalized by R software. For survival analysis of AEBP1, the online tool KM plotter^53^ (http://www.kmplot.com/gastric) was used to detect the prognostic significance of AEBP1 in patients with GC. The KM plotter database includes 876 patients with clinical information from the NCBI GEO database. The survival data were processed online by splitting the patients into two groups according to the best performing threshold, and the results were downloaded from the website. The Kaplan-Meier curves were plotted using GraphPad Prism 5.0 software (GraphPad Software, La Jolla, CA, USA).

The prognostic value of AEBP1 in patients with GC was initially analyzed using the online database OncoLnc (www.oncolnc.org), which included mRNA expression data and survival data of 378 GC patients from The Cancer Genome Atlas (TCGA)-STAD (http://cancergenome.nih.gov/) database^54^. The processed data were downloaded, and Kaplan-Meier curves were plotted using GraphPad Prism 5.0 software.

### Stable short hairpin RNA (shRNA) transfection

The construction of shRNA targeting AEBP1 (sh-AEBP1) and non-targeting control (mock) were performed by GeneChem (Shanghai, China) (Supplementary Table S2). Lentiviral and packaging vectors were cotransfected into HEK293 T cells using Lipofectamine^TM^ 3000 transfection reagent (Invitrogen, Carlsbad, CA, USA) according to the manufacturer’s instructions. Stably transfected cells were selected using 3 μg/mL puromycin (Sigma, USA). The knockdown efficiency of AEBP1 was confirmed by qRT-PCR and Western blotting analysis.

### Colony formation assay

For the colony formation assay, GC cells were seeded in 6-well plates at 500 cells per well in RPMI-1640 containing 10% FBS for 2 weeks until most colonies formed more than 50 cells. After removing the culture medium, colonies were fixed with 4% paraformaldehyde for 30 min and stained with crystal violet for 10 min (Beyotime, Shanghai, China). Only colonies containing more than 50 cells were counted under a light microscope. The experiments were performed in triplicate.

### Cell proliferation assay

The proliferation ability was evaluated using Cell Counting Kit-8 (CCK-8; Dojindo, Kumamoto, Japan) according to the manufacturer’s guidelines. The cells (3000 cells/well) were seeded into 96-well plates and cultured for 24, 48, 72 and 96 h. The CCK-8 reagents were added to each well at the indicated times and were incubated for 1 h. The absorbance was measured with a plate reader at a wave length of 450 nm (Thermo Scientific, USA). The experiments were performed in triplicate.

### Transwell migration and invasion assays

The migratory and invasive capabilities of GC cells were determined using transwell assay (pore size, 8 μm; Corning Incorporated, NY, USA). For the transwell invasion assay, the upper surface of the transwell chambers were precoated with Matrigel (BD Biosciences, Sparks, USA). GC cells were cultured in serum-free RPMI-1640 overnight and were added into the upper chambers (3 × 10^4^ cells for the migration assay and 5 × 10^4^ cells for the invasion assay). For both the migration and invasion assays, the medium in the upper chamber was filled with serum-free RPMI-1640, and the lower chamber was filled with RPMI-1640 medium containing 10% FBS. After incubation at 37 °C for 24 h, cells on the upper surface of the membranes were removed with a cotton swab, and cells on the lower surface of the membranes were fixed with 4% paraformaldehyde for 30 min and stained with crystal violet solution (Beyotime, Shanghai, China) for 10 min. The number of migrated or invaded cells was counted under a microscope from five random fields. All experiments were performed in triplicate.

### Wound healing assay

GC cells (MGC803 and XN0422) were seeded in 6-well plates and were cultured until they reached confluency. Cells were incubated with 10 μg/ml mitomycin C (Sigma Chemical, Inc., St. Louis, MO, USA) for 2 h prior to the scratch assay to inhibit proliferation. A straight scratch was performed with a 200-μL sterile pipette tip. Cells were incubated in serum-free RPMI-1640 medium, and the wound closures were visualized at 0 and 24 h using an Olympus microscope (Olympus IX50; Olympus, Tokyo, Japan). The migration rate (%) was calculated as the percentage of the recovered gap distance compared with the initial wound width. All experiments were performed in triplicate.

### Quantitative real-time PCR (qRT-PCR)

Total RNA was extracted from cell lysates using TRIzol reagent (Invitrogen, CA, USA). Reverse transcription was performed using the Prime Script RT reagent Kit with gDNA Eraser (Takara, Japan). qRT-PCR reactions were performed using SYBR Premix Ex Taq II (Takara, Japan) following the manufacturer’s instructions. The primers used in this study were listed in Supplementary Table S3. The relative mRNA expression levels were normalized against β-actin using the 2^−ΔΔCt^ formula^55^. All experiments were performed in triplicate.

### Western blotting

For total protein extraction, cells were lysed with radioimmunoprecipitation lysis buffer (Beyotime, Shanghai, China) containing phenylmethanesulfonyl fluoride and phosphatase inhibitors. The nuclear proteins were extracted using a Nuclear and Cytoplasmic Protein Extraction Kit (Beyotime, Shanghai, China) according to the manufacturer’s instructions. The protein concentration was measured using a Protein BCA Assay kit (Thermo Fisher Scientific, California, USA). The proteins were separated by electrophoresis in 10% sodium dodecyl sulfate-polyacrylamide gel electrophoresis and were electrotransferred onto polyvinylidene difluoride membranes (Millipore, Billerica, USA). The membranes were blocked with 5% non-fat milk at room temperature for 2 h. Next, the membranes were incubated with primary antibodies against AEBP1 (1:1000, Novus Biologicals, USA), Cyclin D1 (Cell Signaling Technology, CST, Beverly, MA, USA, 1:1000), PCNA (CST, 1:2000), phospho-ERK1/2 (CST, 1:1000), NF-κB p65 (CST, 1:1000), phospho-NF-κB p65 (Ser536) (p-p65) (CST, 1:1000), IκBα (CST, 1:1000), E-cadherin (CST, 1:1000), vimentin (CST, 1:1000), MMP2 (CST, 1:1000), MMP9 (CST, 1:1000), Snail (CST, 1:1000), CXCR4 (Abcam, 1:100), ICAM1 (CST, 1:1000), Lamin B (CST, 1:1000) and β-actin (CST, 1:1000) at 4 °C overnight. The membranes were then incubated with corresponding horseradish peroxidase (HRP)-conjugated secondary antibodies (CST, 1:5000) for 1 h at room temperature. Enhanced chemiluminescence Western blotting detection kits (Bio-Rad, Hercules, CA, USA) were used to visualize the target proteins according to the manufacturer’s instructions. β-Actin was employed as a protein loading control.

### Subcutaneous tumorigenicity and intraperitoneal metastasis assays

Female BALB/c nude mice (5 weeks old) were purchased from the Laboratory Animal Center of the Third Military Medical University (Chongqing, China) and housed in a pathogen-free environment. All animal procedures were approved by the Third Military Medical University Animal Committee and were performed in accordance with the approved University guidelines and regulations. GC cells with different treatments were injected subcutaneously into the axilla of the mice (2 × 10^4^ cells). Subcutaneous tumor growth was inspected every day. The mice were sacrificed at the end of 4 weeks post-implantation, and the xenograft tumors were removed and measured for weight. For intraperitoneal metastasis, GC cells with different treatments were injected intraperitoneally (1 × 10^5^ cells per mouse). After 4 weeks, the mice were sacrificed and numbers of intraperitoneal tumor nodules were counted.

### Statistical analysis

The data were analyzed using Prism 5.0 software (GraphPad Software, La Jolla, CA) and SPSS 19.0 software (IBM SPSS Inc., Chicago, USA). Statistical data were presented as the mean ± standard deviation. Comparisons between two groups were analyzed by Student’s *t* test. Comparison of the IHC score of the tumor and normal tissues was conducted using paired-sample *t* test. Pearson χ^2^ test was used to evaluate the relationship between AEBP1 expression and clinicopathological features in patients with GC. Survival analysis was performed using the Kaplan-Meier method, and survival rates were compared using the log-rank test. Hazard ratios (HRs) and 95% confidence intervals (CIs) were used to estimate the correlation between AEBP1 expression and OS. Cox proportional hazards regression was used for univariate and multivariate survival analyses. Multivariate analysis was performed to identify independent prognostic factors among significantly correlated variables. A value of P < 0.05 was considered significant.

### Data availability

All data generated or analyzed during this study are included in this published article (and its Supplementary Information files).

## Conclusion

In summary, our study illustrates that AEBP1 expression is increased in gastric cancer tissues and cell lines, and elevated expression of AEBP1 predicts poor survival in patients with both early- and late-stage gastric cancer. AEBP1 functions as an oncogene in gastric cancer through promoting cell proliferation, migration and invasion, metastasis and epithelial-mesenchymal transition. Moreover, we demonstrate that AEBP1 promotes epithelial-mesenchymal transition through activating the NF-κB pathway via accelerating the degradation of IκBα. Thus, AEBP1 might be a valuable prognostic marker of patients with gastric cancer and a potential target for treatment.

### Ethics approval and consent to participate

This study was approved by the Ethics Committee of the Southwest Hospital, Third Military Medical University. All procedures performed in studies were in accordance with guidelines of the Helsinki Declaration. Written informed consent was obtained from all patients.

### Availability of data and materials

All statistical analyses were performed using SPSS 19.0. All data analyzed during the present study are included in this manuscript.

## Electronic supplementary material

Supplementary figures and tables
