# Supplementary material for: AEBP1 promotes epithelial-mesenchymal transition of gastric cancer cells by activating the NF-κB pathway and predicts poor outcome of the patients
Source: Sci Rep. 2018 Aug 10;8:11955. doi: 10.1038/s41598-018-29878-6 (PMC6086860; doi:10.1038/s41598-018-29878-6)
Supplement: Supplementary file 1 — Supplementary figures and tables [file 41598_2018_29878_MOESM1_ESM.pdf]

Supplemental Information

AEBP1 promotes epithelial-mesenchymal transition in gastric cancer cells by activating the NF-κB pathway and predicts poor outcome of the patients

Jun-yan Liu[1, #], Lei Jiang[1, 2#], Jia-jia Liu[1], Tao He[1], You-hong Cui[3], Feng Qian[1*], and Pei-wu Yu[1, *]

[1] Department of General Surgery and Center of Minimal Invasive Gastrointestinal Surgery, Southwest Hospital, Third Military Medical University, Chongqing 400038, China

[2] Department of Oncology Surgery, The First Hospital of Lanzhou University, Lanzhou 730030, China

[3] Institute of Pathology and Southwest Cancer Center, Southwest Hospital, Third Military Medical University, Key Laboratory of Tumor Immunopathology of Ministry of Education of China, Chongqing 400038, China

[#] These authors contributed equally to this work.

[*] Correspondence to: Pei-wu Yu M.D. and Feng Qian M.D.

E-mail addresses: yupeiwu0l@sina.com. (Pei-wu Yu); qianfengpuwaia@163.com (Feng Qian).

Key words: gastric cancer, AEBP1, invasion, epithelial-mesenchymal transition, NF-κB

Supplementary Figures:

Supplementary Figure S1:

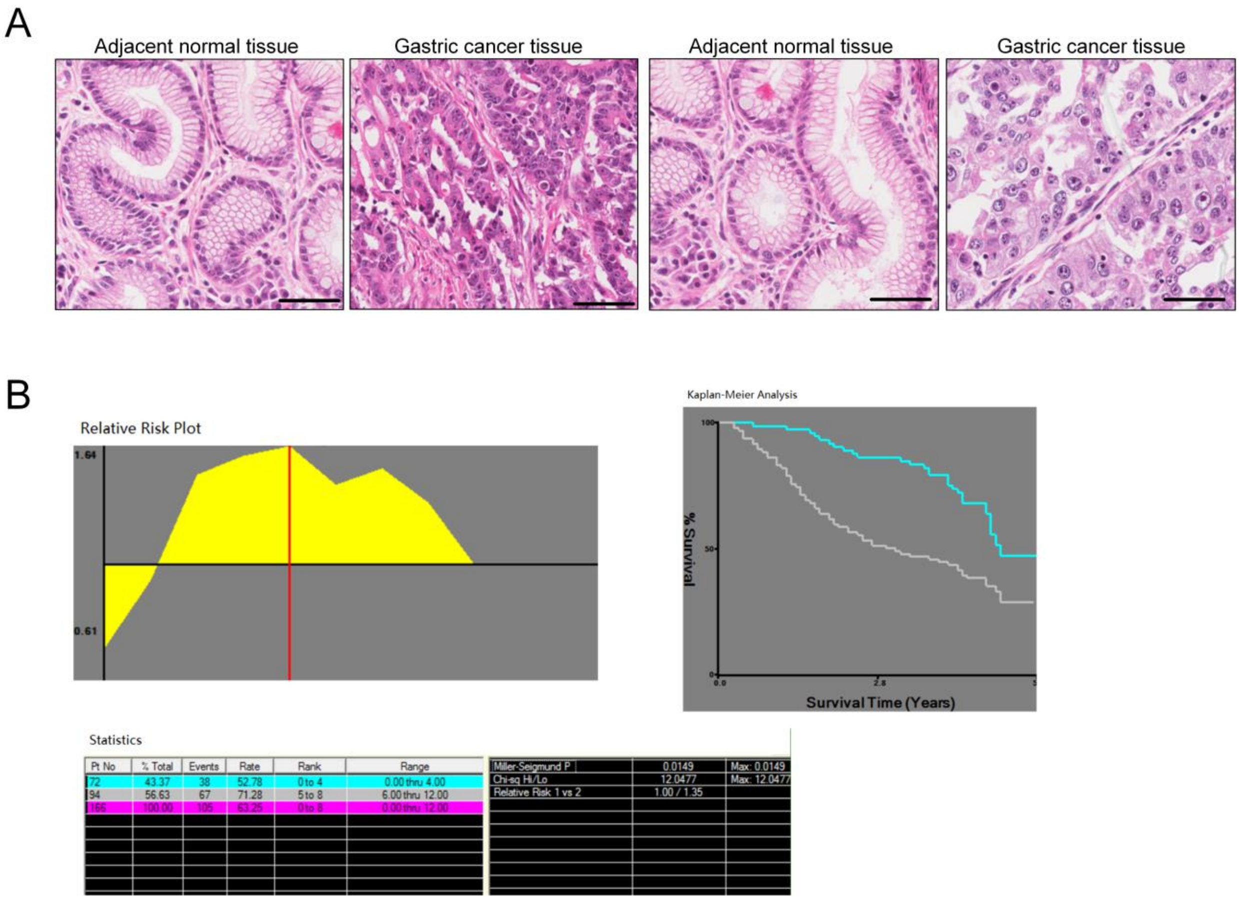

Supplementary Figure S1. H&E staining and the identification of optimal cutoff value using X-tile software. A, Representative H&E images of adjacent normal mucosa and gastric cancer tissue. Bar, 50 μm. B, The relative risk plot of each IHC score generated by X-tile software.

Supplementary Figure S2:

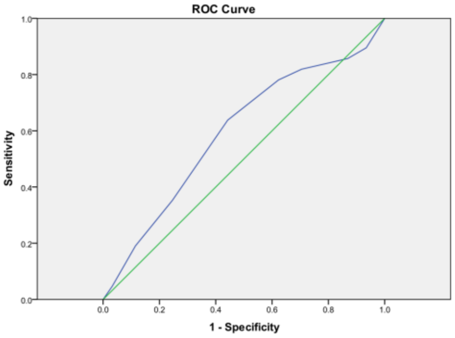

Diagonal segments are produced by ties.

Area Under the Curve

Test Result Variable(s):AEBP1

Area	Std. Error[a]	Asymptotic Sig.[b]	Asymptotic 95% Confidence Interval	
			Lower Bound	Upper Bound
.594	.046	.044	.505	.684

The test result variable(s): AEBP1 has at least one tie between the positive actual state group and the negative actual state group. Statistics may be biased.

a. Under the nonparametric assumption
b. Null hypothesis: true area = 0.5

Coordinates of the Curve

Test Result Variable(s):AEBP1

Positive if Greater Than or Equal To[a]	Sensitivity	1 - Specificity	Youden index
-1.0000	1.000	1.000	.000
.5000	.895	.934	-.039
1.5000	.857	.869	-.012
2.5000	.819	.705	.114
3.5000	.781	.623	.158
5.0000	.638	.443	.195
7.0000	.352	.246	.106
8.5000	.190	.115	.076
10.5000	.048	.033	.015
13.0000	.000	.000	.000

The test result variable(s): AEBP1 has at least one tie between the positive actual state group and the negative actual state group.

a. The smallest cutoff value is the minimum observed test value minus 1, and the largest cutoff value is the maximum observed test value plus 1. All the other cutoff values are the averages of two consecutive ordered observed test values.

Supplementary Figure S2. The identification of optimal cutoff value by Youden's index. The ROC curve and Youden's index generated by SPSS 19.0 software.

Supplementary Figure S3:

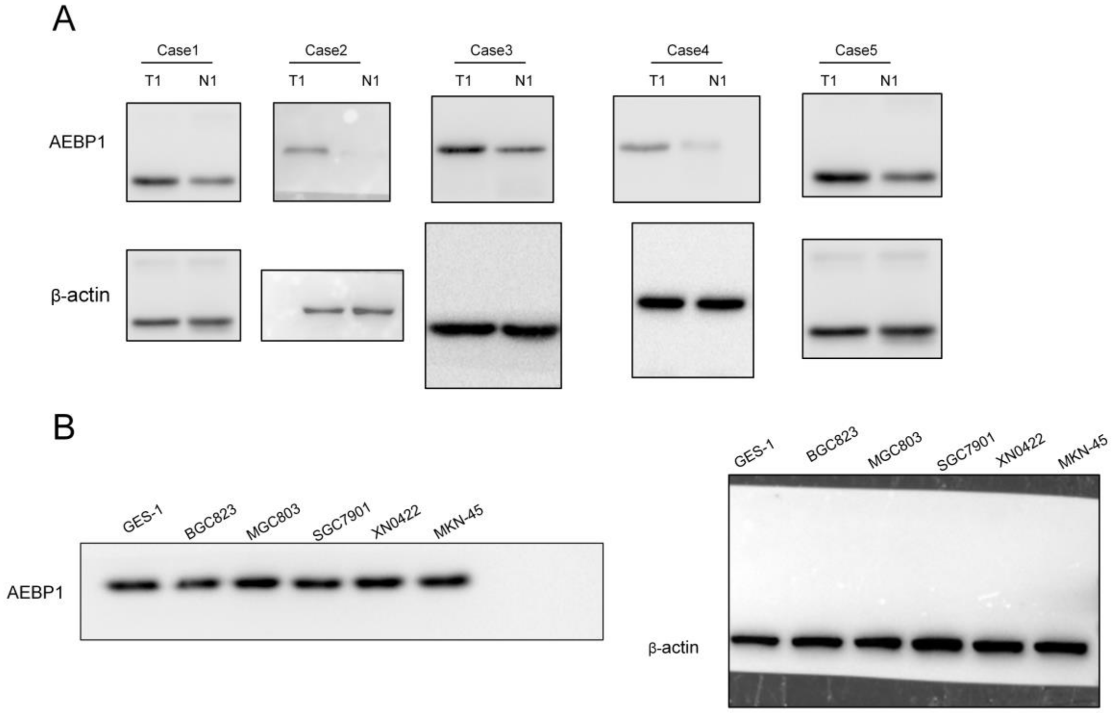

Supplementary Figure S3. A, The full-length western blots of AEBP1 and β-actin expression in 5 paired fresh surgical gastric cancer specimens (tumor, T) and corresponding adjacent normal tissues (normal, N) are shown in the boxed panels (experimental details are described in Methods). The western blots were derived under the same experimental conditions from the same cell lysates. B, The full-length western blots of AEBP1 and β-actin expression in five GC cell lines (MKN-45, BGC823, MGC803, SGC7901 and XN0422) and GES-1 are shown in the boxed panels (experimental details are described in Methods). The western blots were derived under the same experimental conditions from the same cell lysates.

Supplementary Figure S4.

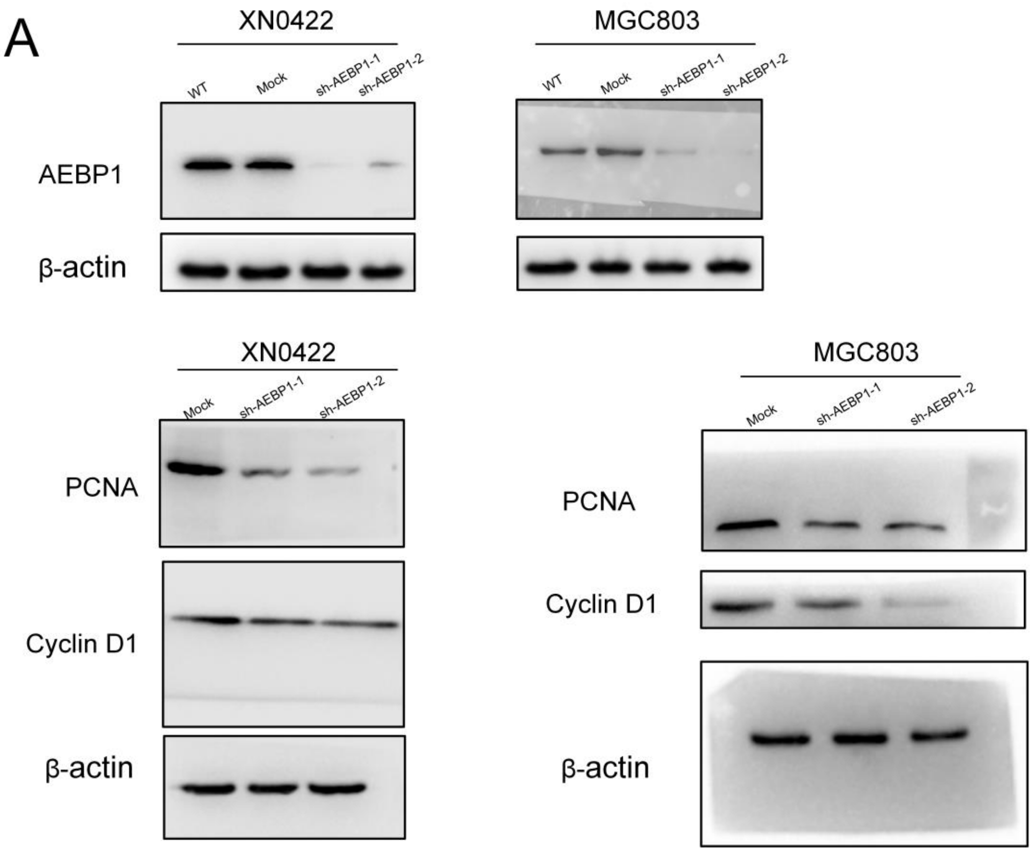

Supplementary Figure S4. A, The full-length western blots of AEBP1 and β-actin expression in MGC803 and XN0422 cells are shown in the boxed panels (experimental details are described in Methods). The western blots were derived under the same experimental conditions from the same cell lysates. WT, wide type; B, The full-length western blots of PCNA, Cyclin D1 and β-actin expression in MGC803 and XN0422 cells are shown in the boxed panels (experimental details are described in Methods). The western blots were derived under the same experimental conditions from the same cell lysates.

Supplementary Figure S5.

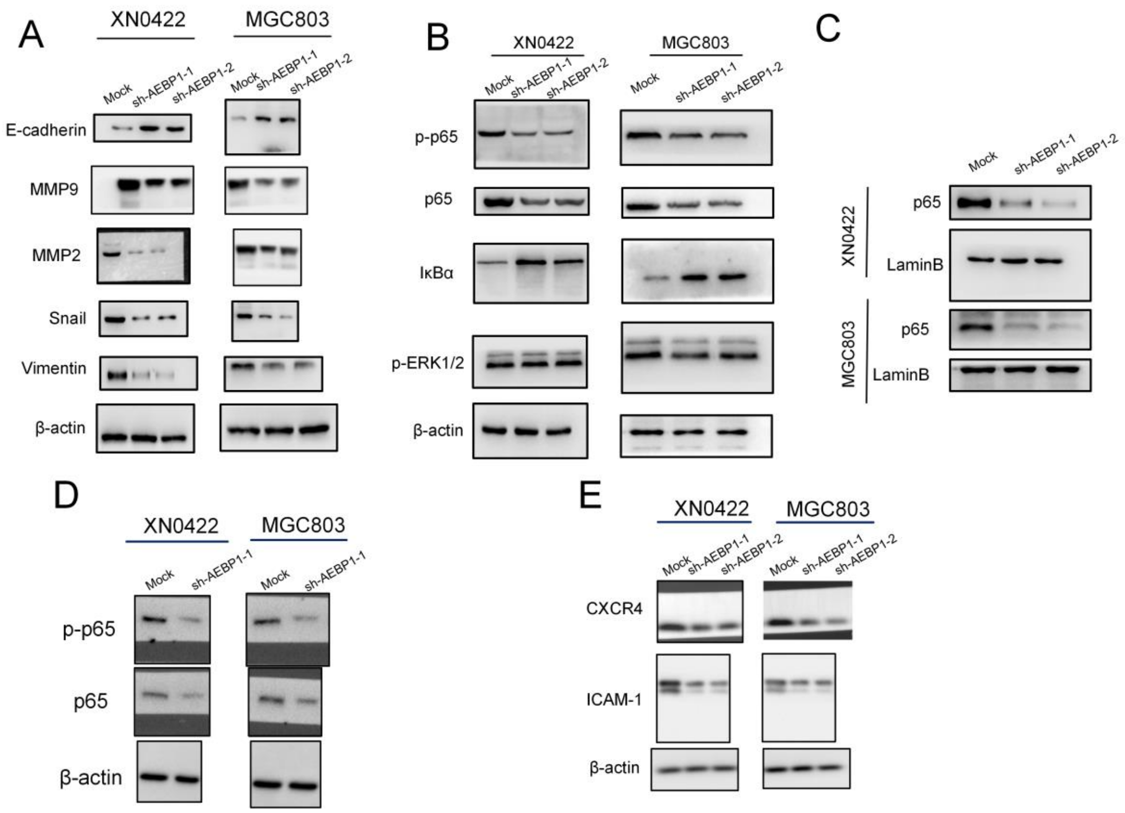

Supplementary Figure S5. A, The full-length western blots of E-cadherin, MMP9, MMP2, snail, vimentin and β-actin expression in MGC803 and XN0422 cells are shown in the boxed panels (experimental details are described in Methods). The western blots were derived under the same experimental conditions from the same cell lysates. B, The full-length western blots of p-p65, p65, IκBα, p-ERK1/2 and β-actin expression in MGC803 and XN0422 cells are shown in the boxed panels (experimental details are described in Methods). The western blots were derived under the same experimental conditions from the same cell lysates. C, The full-length of western blots of nuclear protein expression of NF-κB p65 and Lamin B in MGC803 and XN0422 cells are shown in the boxed panels (experimental details are

described in Methods). The western blots were derived under the same experimental conditions from the same cell lysates. D, The full-length of western blots of p-p65, p65 and β-actin expression of xenograft tumors derived from mock and sh-AEBP1-1 GC cells are shown in the boxed panels (experimental details are described in Methods). The western blots were derived under the same experimental conditions from the same cell lysates. E, The full-length western blots of CXCR4, ICAM1 and β-actin expression in MGC803 and XN0422 cells are shown in the boxed panels (experimental details are described in Methods). The western blots were derived under the same experimental conditions from the same cell lysates.

Supplementary Figure S6.

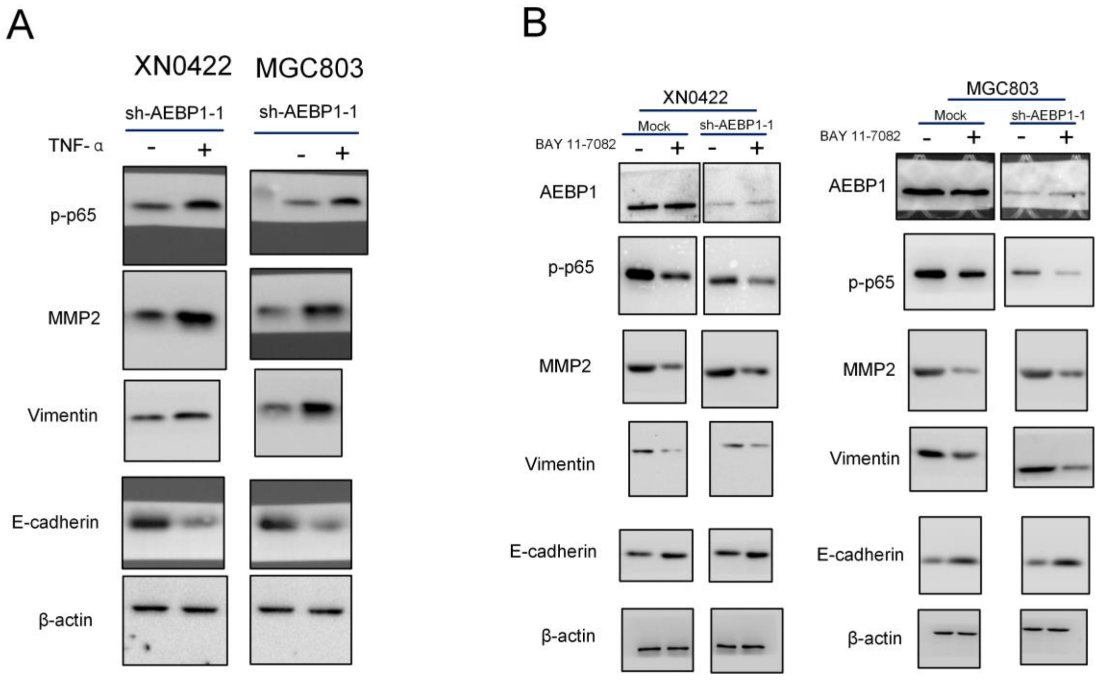

Supplementary Figure S6. A, AEBP1-knockdown XN0422 and MGC803 cells were pretreated with TNF-α or not, the full-length western blots of p-p65, MMP2, vimentin,

E-cadherin and β-actin expression are shown in the boxed panels (experimental details are described in Methods). The western blots were derived under the same experimental conditions from the same cell lysates. B, Mock and sh-AEBP1 treated MGC803 and XN0422 cells were pretreated with BAY 11-708 or not, the full-length of western blots of AEBP1, p-p65, MMP2, vimentin, E-cadherin and β-actin from the lysates of the treated cells are shown in the boxed panels (experimental details are described in Methods). The western blots were derived under the same experimental conditions from the same cell lysates.

Supplementary Tables:

Supplementary Table S1. Patient characteristics

Prognostic variables	Number	Percent (%)
Age		
≥60	63	(37.95)
<60	103	(62.05)
Sex		
Male	113	(68.07)
Female	53	(31.93)
TNM stage		
I	45	(27.11)
II	69	(41.57)
III	43	(25.90)
IV	9	(5.42)
Tumor location		
Proximal	59	(35.54)
Distal	107	(64.46)
Histological grade		
G1	4	(2.41)
G2	44	(26.51)
G3	118	(71.08)
T stage		
T1	24	(14.46)
T2	41	(24.70)
T3	91	(54.82)
T4	10	(6.02)
N stage		
N0	76	(45.78)
N1	37	(22.29)
N2	50	(30.12)
N3	3	(1.81)
M stage		
M0	157	(94.58)
M1	9	(5.42)
AEBP1 expression		
Low	72	(43.37)
High	94	(56.63)

Supplementary Table S2. Sequences of AEBP1 knockdown and mock shRNAs used in this study

Gene		Sequence
sh-AEBP1-1	Forward	5′-CACCGCCAGACATGGGTGATGTACACGAA TGTACATCACCCATGTCTGGC-3′
	Reverse	5′-AAAAGCCAGACATGGGTGATGTACATTCG TGTACATCACCCATGTCTGGC-3′
sh-AEBP1-2	Forward	5′-CACCGCTATGAGGAAATGACCTTTCCGAA GAAAGGTCATTTCCTCATAGC-3′
	Reverse	5′-AAAAGCTATGAGGAAATGACCTTTCTTCG GAAAGGTCATTTCCTCATAGC-3′
Mock	Forward	5′-CCGGTTACGCGTAGCGTAATACGCTCGAG CGTATTACGCTACGCGTAATTTTTG-3′
	Reverse	5′-AATTCAAAAATTACGCGTAGCGTAATACG CTCGAG CGTATTACGCTACGCGTAA-3′

Supplementary Table S3. Sequences of primers used for qRT-PCR in this study

Gene		Sequence
AEBP1	Forward	5′-AGACCACGCCATCTTCCG-3′
	Reverse	5′-CCTTGTTGTTCTCCCACTCG-3′
E-cadherin	Forward	5′-CTACAATGAGCTGCGTGTGG-3′
	Reverse	5′-AGGTCCAGACGCAGGATGGC-3′
Snai1	Forward	5′-GCGCTCTTTCCTCGTCAGG-3′
	Reverse	5′-GGGCTGCTGGAAGGTAAACTCT-3′
MMP2	Forward	5′-CTGGGAGCATGGCGATGGATA-3′
	Reverse	5′-GGAAGCGGAATGGAAACTTG-3′
MMP9	Forward	5′-TTGACAGCGACAAGAAGTGG-3′
	Reverse	5′-GCCATTCACGTCGTCCTTAT-3′
Vimentin	Forward	5′-GACGCCATCAACACCGAGTT-3′
	Reverse	5′-CTTTGTCGTTGGTTAGCTGGT-3′
β-actin	Forward	5′-GAATTCATGTTTGAGACCTTCAA-3′
	Reverse	5′-CCGGATCCATCTCTTGCTCGAAGTCCA-3′
